# Genome-wide profiling of piRNAs in the whitefly *Bemisia tabaci* reveals cluster distribution and association with begomovirus transmission

**DOI:** 10.1371/journal.pone.0213149

**Published:** 2019-03-12

**Authors:** Md Shamimuzzaman, Daniel K. Hasegawa, Wenbo Chen, Alvin M. Simmons, Zhangjun Fei, Kai-Shu Ling

**Affiliations:** 1 U.S. Department of Agriculture, Agricultural Research Service, U.S. Vegetable laboratory, Charleston, SC, United States of America; 2 Boyce Thompson Institute, Cornell University, Ithaca, New York, United States of America; 3 USDA-Agricultural Research Service, Robert W. Holley Center for Agriculture and Health, Ithaca, New York, United States of America; Chinese Academy of Agricultural Sciences, CHINA

## Abstract

The whitefly *Bemisia tabaci* MEAM1 is a notorious vector capable of transmitting many plant viruses, resulting in serious crop loss and food shortage around the world. To investigate potential sRNA-mediated regulatory mechanisms in whiteflies that are affected by virus acquisition and transmission, we conducted small RNA (sRNA) deep sequencing and performed genome-wide profiling of piwi-interacting RNAs (piRNAs) in whiteflies that were fed on tomato yellow leaf curl virus (TYLCV)-infected or non-infected tomato plants for 24, 48, and 72 h. In the present study, piRNA reads ranging from 564,395 to 1,715,652 per library were identified and shown to distribute unevenly in clusters (57 to 96 per library) on the whitefly (*B*. *tabaci* MEAM1) genome. Among them, 53 piRNA clusters were common for all treatments. Comparative analysis between libraries generated from viruliferous and non-viruliferous whiteflies identified five TYLCV-induced and 24 TYLCV-suppressed piRNA clusters. Approximately 62% of piRNAs were derived from non-coding sequences including intergenic regions, introns, and untranslated regions (UTRs). The remaining 38% were derived from coding sequences (CDS) or repeat elements. Interestingly, six protein coding genes were targeted by the TYLCV-induced piRNAs. We identified a large number of piRNAs that were distributed in clusters across the whitefly genome, with 60% being derived from non-coding regions. Comparative analysis revealed that feeding on a virus-infected host caused induction and suppression of only a small number of piRNA clusters in whiteflies. Although piRNAs primarily regulate the activity of transposable elements, our results suggest that they may have additional functions in regulating protein coding genes and in insect-virus interactions.

## Introduction

Small RNAs (sRNAs) are 19–31 nucleotide (nt) non-coding regulatory elements commonly found in plants, animals, including insects [1–4]. They play important roles in regulating gene expression, transposable elements (TE), or parasite immunity [2,5–7]. Major classes of sRNAs include small interfering RNAs (siRNAs) and microRNAs (miRNAs), which are primarily involved in silencing gene expression [1,2]. Another class of sRNAs known as PIWI-interacting RNAs (piRNAs) has been identified in vertebrate and invertebrate animals [4,5,8,9]. These piRNAs ranging from 26 to 31 nt are considered to regulate the activity of transposable elements as well as to silence the expression of protein coding genes [9–11].

The biogenesis of piRNAs has been studied in fruit fly (*Drosophila melanogaster*), honey bee (*Apis melifera*), mosquito (*Anopheles gambiae*), and other insects [4,11–13]. Three core proteins known as P-element induced wimpy testis (Piwi), Aubergine (Aub) and Argonaute-3 (Ago3) are required for piRNA biogenesis [7,8,12]. piRNAs are generated from distinct chromosomal regions referred to as piRNA clusters [7,8] and can vary in size from 1 kb to 250 kb and may contain as few as 10 to many thousands of piRNAs [13–15]. Two mechanisms of piRNA biogenesis has been described in *Drosophila* and other insects [7,13,14]. In first mechanism, long single-stranded piRNA precursors in the sense or antisense orientation are transcribed from piRNA clusters, and then processed into primary piRNAs by the cytoplasmic endonuclease, Zucchini (Zuc) [8,16,17]. The primary piRNAs, in association with Piwi proteins, will then form a RNA-induced silencing complex (RISC), which leads to piRNA-directed cleavage of mRNAs [8,14]. In the second mechanism, secondary piRNAs are generated through an amplification loop cycle, referring to as the ping-pong cycle [13,14]. Here, the target transcripts are cleaved by Aub-piRISCs, resulting in 5′- cleaved products being released and degraded in the process, whereas 3′- cleaved products are loaded onto Ago3 and subsequently processed into secondary piRNAs [13,18]. The hallmarks of the ping-pong mediated amplification of piRNAs are evidenced with a strong uridine (U) bias at the 5’ end for Aub-associated piRNAs and an adenosine (A) at position 10 for Ago3-associted piRNAs [7,8,13]. A 10 base-pair overlap is often seen between the complementary primary and secondary piRNAs.

The functions of piRNAs are highly conserved among a number of animal and insect species [5,8,9,12,13], which are primarily involved in silencing the activity of transposable elements (TEs) in germline cells but are also active in other cell types [4,7,8,19,20]. TEs represent an important part of repetitive elements and can drive intra-genomic variation and genome diversification. The movement of TEs from one part of a genome to another occurs via an RNA or DNA intermediate, and thus, are classified as retrotransposons (class I) and DNA transposons (class II), respectively [21,22]. The Class I TEs are composed of long terminal repeat (LTR) and non-LTR (NLTR) retrotransposons. Families of LTR retrotransposons, including gypsy, copia and pao, are abundant in diverse insect species [7,11,23]. Similarly, NLTR, including long interspersed nuclear elements (LINEs) and short interspersed nuclear elements (SINEs), are also well studied transposable elements in insects [8,13]. On the other hand, the Class II DNA transposons, including maverick, mariner, hAT, harbinger, and P elements, are also abundant in insects [7,13,24]. Thus, piRNAs can regulate the activity of both retrotransposons and DNA transposons. Furthermore, some piRNAs are non-repetitive and non-transposon-related, which can regulate expression of protein-coding genes [8,13]. In addition to the functions of piRNAs in controlling TE activities and silencing the expression of protein-coding genes, the piRNA pathway has also been shown to be involved in antiviral response in several insects (*D*. *melanogaster*, *A*. *gambiae* and *A*. *aegypti* [6,7,25,26]. Altogether, piRNAs play diverse roles in the pathways associated with insect development, reproduction and immune responses.

The whitefly *Bemisia tabaci* Middle-East Asia Minor 1 (MEAM1) is one of the most widely distributed whitefly cryptic species, which is capable of transmitting hundreds of plant viruses to cause devastating losses on important agricultural crops, including beans, cassava, cotton, melon, sweetpotato and tomato [27,28]. Whitefly-transmitted viruses are classified in the genera of *Begomovirus*, *Crinivirus*, *Ipomovirus*, *Torradovirus* and *Carlavirus*. One of the most important begomoviruses is tomato yellow leaf curl virus (TYLCV), which has also been the focus for numerous other studies to understand the whitefly-begomovirus interactions [29–31].

In the present study, our preliminary analysis of sRNA sequence datasets revealed an enrichment of sRNAs (ranging from 26–31 nt) in addition to the typical miRNAs, which led us to explore the piRNA profiling. We performed genome-wide identification of piRNA clusters in whitefly (*B*. *tabaci*, MEAM1) and analyzed their expression levels in whiteflies feeding on TYLCV-infected or uninfected tomato plants for 24, 48, and 72 h. With ~45% of the whitefly genome sequence comprised of repeat elements, including miniature inverted repeat transposable elements (MITEs), LINEs, SINEs, LTRs and DNA transposons [28], piRNAs, involved in regulation of transposable elements, may play an important role in maintaining whitefly genome integrity, reproduction and antiviral immune responses. The piRNA pathway has been linked to other epidemiologically important phenotypes in mosquitoes [20,26,32]. The role of piRNAs in antiviral immune responses in both *Aedes aegypti* and *A*. *albopictus* has also been reported by multiple research groups [20,25,26]. Knowledge of the mechanism on how the piRNA pathway regulates reproduction and antiviral immune responses in whitefly could be useful for both fundamental and applied research. Our enhanced understanding of the piRNA pathway in whitefly could facilitate the identification of novel targets for vector control using RNAi and genome editing technologies.

## Materials and methods

### Whiteflies and feeding assays

The same whitefly materials under the identical treatments used in our previous transcriptome analysis [31] were used in the present study. Briefly, a whitefly *B*. *tabaci* MEAM1 colony was maintained at the USDA-ARS, U.S. Vegetable Laboratory on broccoli (*Brassica oleracea* L. var. *botrytis*) in a greenhouse (26°C ± 5°C). The MEAM1 colony was confirmed using PCR primers to amplify the mitochondrial cytochrome oxidase 1 gene [19]. Approximately 1,500 adult whiteflies were transferred to TYLCV-infected or uninfected tomato (*Solanum lycopersicum* cv. Moneymaker) plants and were allowed to feed for 24, 48, or 72 h, as described by Hasegawa et al. [31]. At the end of each time point, 200–500 living whiteflies were collected and immediately stored at -80°C until processing. Three biological replicates were performed for each treatment. Those viruliferous whiteflies were demonstrated in our previous study to contain TYLCV in acquisition periods of 24, 48 and 72 h, whereas those whiteflies fed on non-infected tomato plants were negative for TYLCV [31].

### RNA isolation and library preparation

Total RNA was isolated with a TRIzol Reagent (Thermo Fisher Scientific, USA) according to the manufacturer’s protocol, and purified with the Direct-zol RNA MiniPrep kit (Zymo, USA). sRNAs were then polyacrylamide gel electrophoresis (PAGE)-purified and used to construct sRNA libraries as described [33,34]. A total of 18 sRNA libraries were constructed and sequenced on an Illumina HiSeq 2500 system.

### Processing of sRNA raw reads

Raw sRNA reads were processed to remove adapters and low quality sequences using an in-house perl script included in the VirusDetect package [35]. For piRNA analysis, reads shorter than 25 nt and longer than 40 nt were also removed. The remaining size-selected reads were aligned to the Rfam database (Version 12.1) (http://rfam.sanger.ac.uk/) to filter out any reads that were mapped to rRNAs, tRNAs, and snRNAs. Raw and processed sequencing data are available in the GEO (Gene Expression Omnibus) database of the National Center for Biotechnology Information (NCBI) under accession number GSE111343.

### Identification of piRNA clusters in whitefly

The high-quality cleaned sRNA reads were aligned to the whitefly reference genome using Bowtie [36] allowing up to two mismatches. Replicates were combined and genome-mapped reads were used to identify piRNA clusters using the proTRAC (Version 2.3) pipeline [37] with default parameters. proTRAC predicts potential piRNAs and piRNA clusters based on typical characteristics such as the number of loci with uridine (U) at position 1 or adenosine (A) at position 10, the number of loci within the typical piRNA length (26–31 nt), strand bias, and sliding window of 5 kb. Whitefly gene annotation and repeat sequence annotation obtained from the whitefly genome database [28] were utilized while running the proTRAC pipeline. The proTRAC output provides the normalized quantitative expression of piRNA clusters in reads per million mapped reads (RPM). This pipeline also generates a fasta file for individual clusters that contains all piRNA sequences. Differentially expressed piRNA clusters at each time points were identified using edgeR [38], with a cutoff of adjusted p-value < 0.05. In addition, we calculated the overall percentage for sense versus antisense strand derived piRNAs, and single versus multiple loci derived piRNAs.

### piRNA cluster associated genes and transposable elements

Genes and repeat elements mapped to the piRNA clusters were retrieved from proTRAC output. We calculated the percentage of CDS-, repeat elements- and non-coding sequence-derived piRNAs utilizing the piRNA fasta files generated by proTRAC. Repeat annotation from the whitefly genome database [28] was utilized to divide piRNA cluster mapped repeat elements into different classes and families. We identified commonly expressed piRNA clusters across all time points using bedtools intersect [39]. Similarly, piRNA clusters that were induced or suppressed in whiteflies fed on uninfected versus TYLCV-infected plants were identified.

## Results

### Sequencing and initial processing of sRNA reads

**Fig 1:**
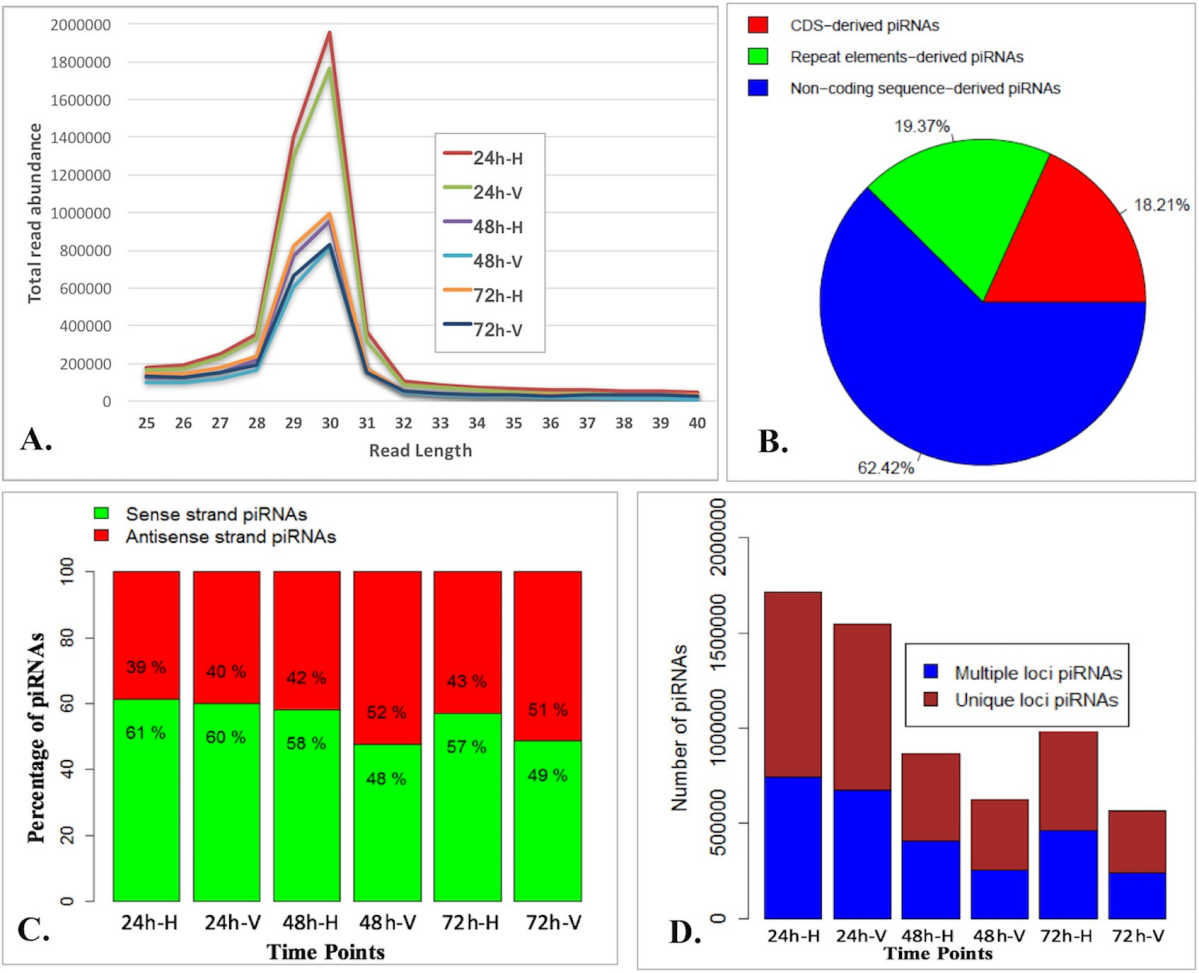
**A. Distribution of sRNA read lengths.** Size distribution of sequence reads with peaking at 28–31 nt which is usual size range for piRNAs. Horizontal axis provides read length and vertical axis provides total number of reads at specific length. 24 h-H, 48 h-H, and 72 h-H represents whiteflies fed on uninfected tomato for 24, 48 and 72 hours, respectively. 24 h-V, 48 h-V, and 72 h-V represents whiteflies fed on TYLCV-infected tomato for 24, 48 and 72 hours, respectively. **Fig 1B.** Distribution of piRNAs in different genomic regions. Percentage of all identified piRNAs mapping to different genomic features. Red color denotes piRNAs derived from coding sequences (CDS), green color denotes piRNAs derived from repeat elements, and blue denotes piRNAs derived from non-coding sequences including intergenic regions, introns and UTRs. **C).** Distribution of piRNAs based on strand. Green color denotes percentage of piRNAs derived from sense strand whereas brown color denotes percentage of piRNAs derived from antisense strand. **D).** Distribution of piRNAs based on mapping to number of genomic loci. Blue color denotes piRNAs mapped to multiple genomic loci whereas brown color denotes piRNAs mapped to single unique genomic loci. 24 h-H, 48 h-H, and 72 h-H represents whiteflies fed on uninfected tomato for 24, 48 and 72 hours, respectively. 24 h-V, 48 h-V, and 72 h-V represents whiteflies fed on TYLCV-infected tomato for 24, 48 and 72 hours, respectively.

To shed light on the whitefly piRNA profiles in response to feeding on TYLCV-infected tomato, deep sequencing of sRNAs was performed using RNAs isolated from whiteflies fed on either TYLCV-infected or uninfected tomato for 24, 48, and 72 h. We performed three biological replicates for each time point. Sequencing of 18 sRNA libraries produced a total of approximately 175 million reads with a range of approximately 5 to 13 million reads per library (S1 Table) and Pearson correlation coefficient (S2 Table) indicates high reproducibility of sequencing libraries. After trimming and removing low quality reads, rRNAs, tRNAs, and snRNAs, we found that reads with 28–31 nt in length were the most abundant in the datasets (Fig 1A), indicating that the libraries were enriched for reads corresponding to piRNAs. Reads that had lengths within the range of expected piRNAs of 26–40 nt were selected for further analysis.

### Expression of core components of piRNA pathway genes in *Bemisia tabaci*

Core components of piRNA pathway genes, Ago-3 (Bta04637), Aub (Bta08949), and piwi (Bta00007 and Bta00198) are conserved in the *B*. *tabaci* genome [28]. We retrieved the expression values of the above genes from a recently published transcriptome dataset [31] that was generated using the same pool of total RNA from which sRNAs were isolated. We found that all core component genes of the piRNA pathway were expressed at all three time points (Table 1). The expression of *Ago-3* ranged in16 to 18 RPKM, and *Aub* in 83 to 93 RPKM. Both piwi genes were also expressed, although at lower levels (Table 1), indicating a functional piRNA pathway is present in the whitefly *B*. *tabaci*. P-values (Table 1) obtained from edgeR [38] analysis indicate that expression level of these piRNA pathway genes did not change significantly across all three time points.

**Table 1:** Expression of genes encoding the core components of piRNA pathways.

Gene	Annotation	24 h-H	24 h-V	p-val	48 h-H	48 h-V	p-val	72 h-H	72 h-V*	p-val
Bta04637	argonaute-3	18.38	16.28	1	16.94	17.42	1	17.13	16.09	1
Bta08949	aubergine-like protein	90.05	88.42	1	90.18	97.91	1	93.21	83.52	1
Bta00007	Piwi-like protein	5.8	4.62	0.97	4.69	4.43	1	5.15	5.13	1
Bta00198	Piwi-like protein	2.85	2.51	1	3.47	2.68	1	3.42	3.46	1

*24 h-H, 48 h-H, and 72 h-H represents whiteflies fed on uninfected tomato for 24, 48 and 72 hours respectively. 24 h-V, 48 h-V, and 72 h-V represents whiteflies fed on TYLCV-infected tomato for 24, 48 and 72 hours respectively. Expression values are provided in reads per kilobase per million mapped reads (RPKM). Expression values and edgeR [38] calculated p-values were retrieved from our recently published article [31].

### Identification of piRNA clusters

Whitefly genome-mapped reads were used to identify piRNA clusters using proTRAC pipeline [37]. We detected 57 to 96 clusters of piRNAs at each of the three different time points and treatments (Table 2 and S2 Table). The total number of piRNAs at each time point ranged from 564,395 to 1,715,652 (Table 2). The results obtained from the proTRAC pipeline also provides a detailed graphical view of each cluster. A representative view of an identified graphical cluster was shown in S1 Fig. On average, each cluster contained 13,998 piRNAs. We found piRNA clusters are derived from either positive or negative strand (S2 Table), suggesting they are mono directionally transcribed. The percentage of sense strand-derived piRNAs gradually decreased (from 60% to 49%) over the three tested time periods with acquisition of TYLCV, whereas the antisense strand-derived piRNAs increased (from 40% to 51%) over the same period time (Fig 1C). Additionally, we identified piRNAs matching to single genomic loci as well as multiple genomic loci. The percentage of piRNAs matching to a single genomic loci ranged from 41% to 47% whereas the percentage of piRNAs matching to multiple genomic loci ranged from 53% to 59% (Fig 1D). When mapped to the whitefly *B*. *tabaci* MEAM1 genome, piRNAs were shown to be derived from either coding sequences (CDS), transposable elements, or non-coding sequences. We found that ~ 62% of whitefly piRNAs were derived from non-coding sequences, which included intergenic regions, introns, and UTRs (Fig 1B). The potential roles for non-coding sequence derived piRNAs are still unknown. Approximately 38% of piRNAs were derived from CDS and repeat elements (Fig 1B).

**Table 2:** Summary of identified piRNA clusters in different libraries.

Library*	Number of piRNA clusters	Total piRNAs
24 h-H	96	1,715,652
24 h-V	92	1,547,894
48 h-H	68	868,509
48 h-V	67	623,211
72 h-H	71	993,629
72 h-V	57	564,395

*24 h-H, 48 h-H, and 72 h-H represents whiteflies fed on uninfected tomato for 24, 48 and 72 hours respectively. 24 h-V, 48 h-V, and 72 h-V represents whiteflies fed on TYLCV-infected tomato for 24, 48 and 72 hours respectively.

### CDS and repeat elements derived piRNAs

Apart from the non-coding genomic-region derived piRNAs, there was a significant percentage of piRNAs that were derived from gene coding sequences and repeat elements which were of major interests for this study. The percentage of CDS-derived piRNAs in whiteflies fed on uninfected tomato were 14.6%, 21.0%, and 19.6% of all piRNAs for 24 h, 48 h and 72 h, respectively, whereas the percentages were 15.0%, 22.6%, and 26.3% in whiteflies fed on TYLCV-infected tomato (S2 Fig). These piRNAs were derived from genes that were predicted to be involved in diverse growth, developmental and metabolic pathways (S3 Table).

piRNAs are well known for their involvement in silencing the activity of transposable elements. Overall 19.4% of all identified piRNAs were derived from different classes of repeat elements (Fig 1B and S4 Table). For whiteflies fed on uninfected and TYLCV-infected tomato for 24 h, approximately 16% of piRNAs were derived from repeat elements, whereas at 48 h, it increased to ~ 22% (S2 Fig). Similarly, we found approximately 21% and 23% of piRNAs derived from repeat elements at 72 h for whiteflies fed on uninfected and TYLCV-infected tomato, respectively (S2 Fig).

**Fig 2:**
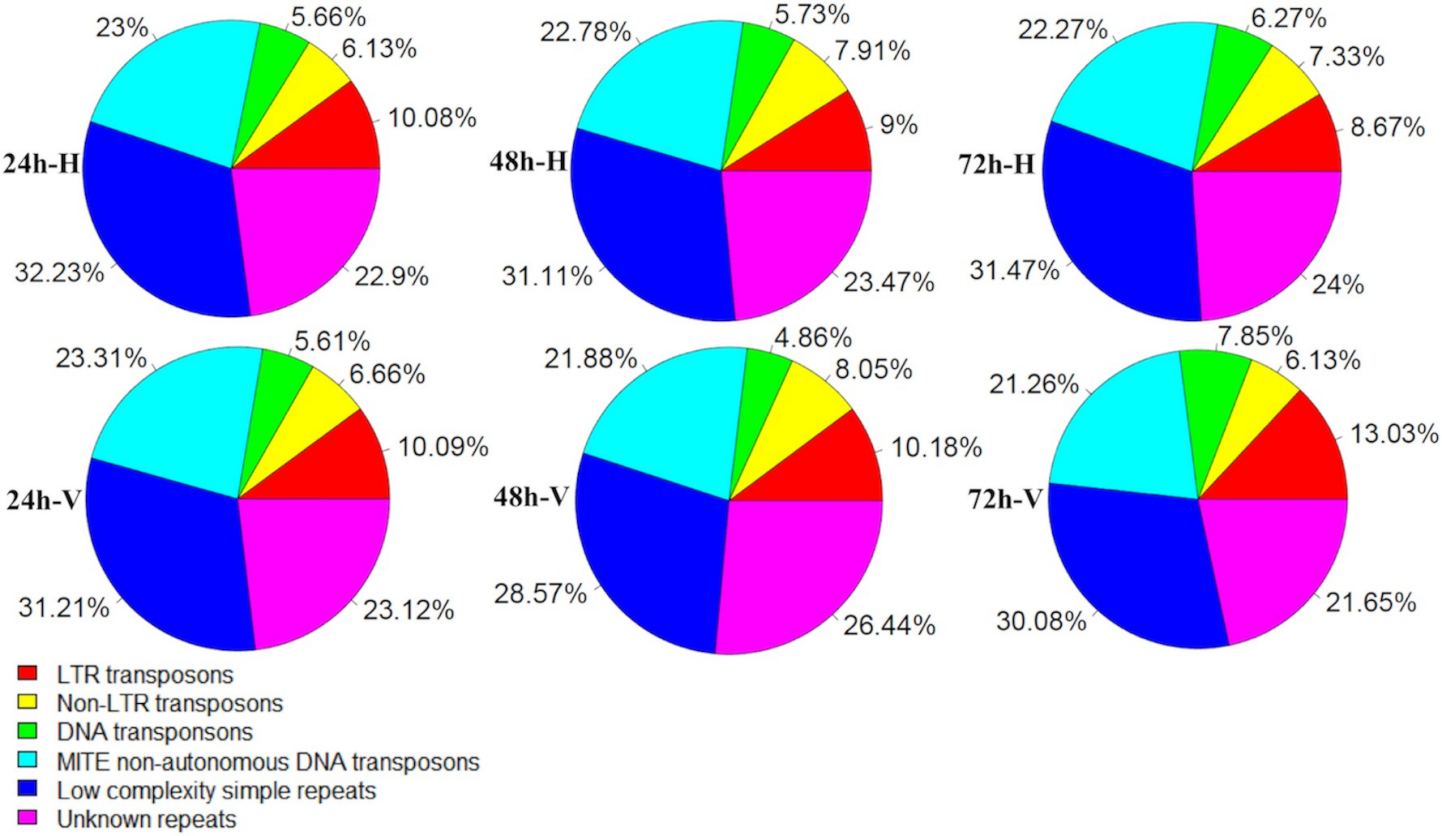
Distribution of repeat elements in piRNA clusters. Different color denotes different families of transposable elements. It shows the percentage of specific repeat family present at specific time points after whiteflies fed on uninfected/TYLCV-infected tomato. 24 h-H, 48 h-H, and 72 h-H represents whiteflies fed on uninfected tomato for 24, 48 and 72 hours, respectively. 24 h-V, 48 h-V, and 72 h-V represents whiteflies fed on TYLCV-infected tomato for 24, 48 and 72 hours, respectively.

Further analysis of repeat elements associated with piRNA clusters provided a comprehensive view of silencing of both class I (retrotransposons) and class II (DNA transposons) transposable elements. Two major families of retrotransposons are LTR and non-LTR retrotransposons. We found approximately 10%, 9% and 8% of LTR repeat elements were targeted by piRNA clusters at 24 h, 48 h, and 72 h, respectively, in whiteflies fed on uninfected tomato (Fig 2). Similarly, in whiteflies fed on TYLCV-infected tomato, we found 10%, 10%, and 13% of LTR repeat elements were targeted by piRNA clusters at 24 h, 48 h, and 72 h, respectively (Fig 2). We also identified piRNA clusters associated with non-LTR repeat elements that included LINEs and SINEs for each time point. Overall approximately 6–8% of non-LTR repeat elements were targeted by piRNA clusters across all time points. Our analysis identified a small percentage of DNA transposons targeted by piRNA clusters. They ranged between 4% and 7% of the total repeat elements targeted by piRNA clusters at all time points (Fig 2). Additionally, we found a major class of repeat elements targeted by piRNA clusters, called MITE elements, which ranged from 21% to 23% of the total repeat elements targeted by piRNA clusters across three different time points (Fig 2).

### Comparison of identified piRNA clusters across three time points

To identify common and treatment-specific piRNA clusters, we performed comparative analysis across all time points using genomic coordinates of the identified piRNA clusters. We found 53 clusters that were commonly expressed across all time points in whiteflies fed on TYLCV-infected or uninfected tomato, and had normalized expression values that ranged from a few hundred to a few thousand RPM (S5 Table). We performed statistical test using edgeR to determine differentially expressed piRNA clusters at three time points separately. P-values (S5 Table and Table 3) obtained from edgeR analysis indicate that one of these common piRNA clusters showed significant differential expression. The top 10 highly expressed piRNA clusters were expressed at 2,606 to 10,708 RPM (Table 3) and the edgeR obtained p-values are greater than 0.05.

**Table 3:** Top 10 highly expressed piRNA clusters across three time points.

piRNA Cluster#	Genomic Coordinate	24 h-H	24 h-V	p-val	48 h-H	48 h-V	p-val	72 h-H	72 h-V*	p-val
Cluster 14	Scaffold1496: 970038–988027	8653.81	8785.46	0.9998	6585.59	6929.98	0.5533	7327.08	6488.66	0.1387
Cluster 51	Scaffold942: 2258000–2294025	10483.82	10708.49	0.9998	4817.16	5109.44	0.5533	8611.14	5167.09	0.1387
Cluster 6	Scaffold12176: 1–8412	5176.25	5281.12	0.9998	3769.10	3932.87	0.5533	4396.97	3952.82	0.1387
Cluster 34	Scaffold2737: 1425031–1439998	5036.33	5075.96	0.9998	4287.83	4512.69	0.5533	4445.80	3819.97	0.4808
Cluster 16	Scaffold1558: 1069000–1080144	4824.27	4932.09	0.9998	3633.06	3849.60	0.5533	4179.38	3796.12	0.1387
Cluster 40	Scaffold3872: 103000–128026	4776.27	4861.66	0.9998	3710.97	3915.16	0.5533	4157.38	3639.44	0.1387
Cluster 44	Scaffold637: 935001–951025	4626.02	4675.21	0.9998	3745.70	3887.18	0.5533	4018.03	3585.03	0.1387
Cluster 50	Scaffold942: 760005–780924	4132.76	4153.56	0.9998	3403.43	3748.42	0.5533	3576.29	2941.60	0.1387
Cluster 31	Scaffold2605: 3026002–3046017	3315.15	3469.88	0.9998	3052.21	3023.40	0.5533	3100.27	2910.32	0.163
Cluster 25	Scaffold2124: 493014–515027	4412.40	4478.17	0.9998	2643.39	2741.01	0.5533	3676.77	2606.07	0.5941

#Common cluster are named sequentially starts from 1 to 53. P-values were calculated using a Bioconductor package edgeR [38].

*24 h-H, 48 h-H, and 72 h-H represents whiteflies fed on uninfected tomato for 24, 48 and 72 hours respectively. 24 h-V, 48 h-V, and 72 h-V represents whiteflies fed on TYLCV-infected tomato for 24, 48 and 72 hours respectively. Expression values are provided in reads per million mapped reads (RPM).

### Induced piRNA clusters in whitefly acquired TYLCV

**Fig 3:**
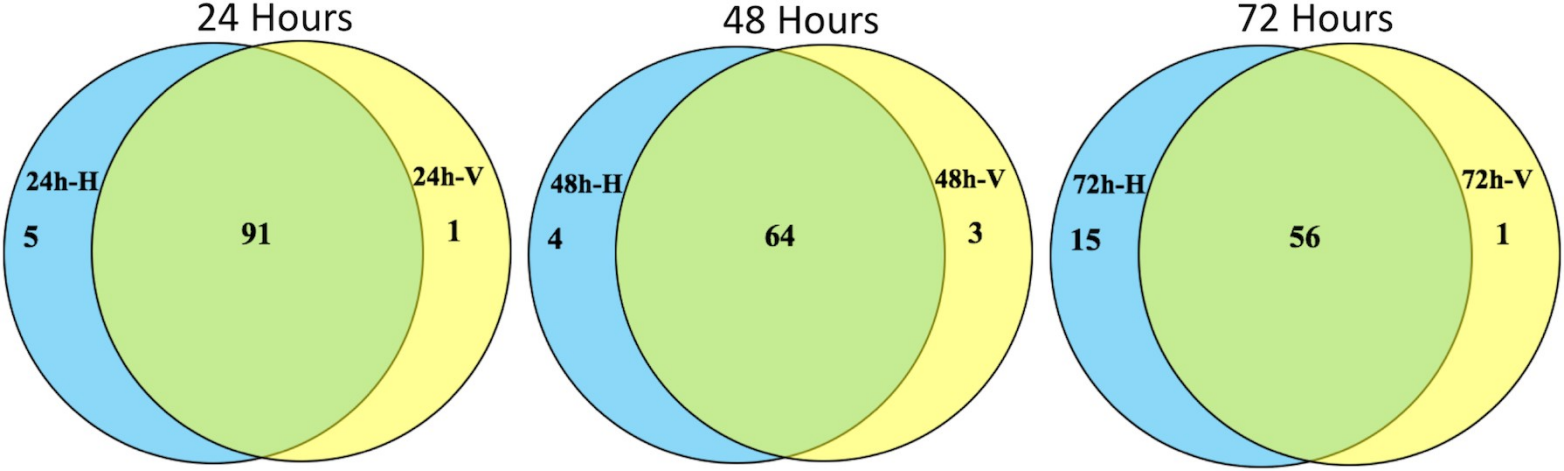
Comparison of identified piRNA clusters across three time points. Most piRNA clusters are common, and only handful of them are induced in whiteflies fed on TYLCV-infected tomato. 24 h-H, 48 h-H, and 72 h-H represents whiteflies fed on uninfected tomato for 24, 48 and 72 hours, respectively. 24 h-V, 48 h-V, and 72 h-V represents whiteflies fed on TYLCV-infected tomato for 24, 48 and 72 hours, respectively.

To evaluate whether the expression of certain piRNA clusters may be affected upon TYLCV acquisition, we identified five piRNA clusters that were exclusively induced and 24 piRNA clusters that were suppressed in whiteflies fed on TYLCV-infected tomato (Fig 3 and Table 4). Low p-values obtained from edgeR analysis indicate these expression differences are significant. Among those five piRNA clusters that were induced in whiteflies when fed on TYLCV-infected tomato (Table 4), their expression ranged from 592 to 2,387 RPM and targeted six protein coding genes as well as a number of transposable elements (Tables 5 and 6). Three of the protein coding genes (Bta07031, Bta05801, Bta02620) have unknown functions, while the other three genes (Bta07186, Bta07187, Bta07188) were annotated as encoding a COMM domain-containing protein, a sentrin-specific protease, and a mitochondrial carrier protein. In addition, these induced piRNA clusters targeted both class I retrotransposons and class II DNA transposons. The piRNA targets included multiple members of different families of transposable elements (Table 6).

**Table 4:** Primary piRNA clusters induced or suppressed upon TYLCV acquisition.

piRNA Cluster*	Genomic Coordinates	Cluster Size (bp)	Normalized reads (RPM)	P-value
**Induced piRNAs in viruliferous whiteflies**				
24 h-V_Cluster 56	Scaffold27: 2717004–2735011	18008	2387.56	3.35E-62
48 h-V_Cluster 27	Scaffold1805: 1007000–1012027	5028	594.65	8.8E-50
48 h-V_Cluster 42	Scaffold27: 2727003–2734958	7956	727.63	1.34E-47
48 h-V_Cluster 57	Scaffold699: 1484000–1489026	5027	592.92	2.21E-51
72 h-V_Cluster 9	Scaffold1335: 74003–81015	7013	764.65	1.91E-55
**Suppressed piRNAs in viruliferous whiteflies**				
24 h-H_Cluster 32	Scaffold16276: 8857001–8864026	7026	865.45	2.5E-58
24 h-H_Cluster 39	Scaffold1805: 1007000–1013011	6012	736.44	2.47E-54
24 h-H_Cluster 43	Scaffold2068: 132008–140016	8009	1007.06	8.74E-56
24 h-H_Cluster 49	Scaffold226: 4092002–4102027	10026	2289.33	3.35E-62
24 h-H_Cluster 72	Scaffold469: 1514001–1520967	6967	941.28	5.89E-55
48 h-H_Cluster 21	Scaffold1580: 896006–902026	6021	640.28	2.8E-48
48 h-H_Cluster 36	Scaffold226: 4014011–4070020	56010	23289.99	2.58E-86
48 h-H_Cluster 38	Scaffold2418: 1–5665	5665	3247.88	4.64E-63
48 h-H_Cluster 57	Scaffold653: 1–7998	7998	981.35	8.35E-55
72 h-H_Cluster 3	Scaffold1144: 774002–780020	6019	676.32	1.37E-40
72 h-H_Cluster 9	Scaffold130: 3650002–3656961	6960	879.15	6.31E-49
72 h-H_Cluster 13	Scaffold137: 1429000–1439844	10845	2168.71	4.19E-59
72 h-H_Cluster 17	Scaffold147: 9362004–9370728	8725	1354.55	1.45E-52
72 h-H_Cluster 22	Scaffold16276: 8857001–8863024	6024	662.55	3.92E-47
72 h-H_Cluster 30	Scaffold199: 5961009–5973027	12019	1953.23	3.01E-56
72 h-H_Cluster 36	Scaffold226: 4014011–4070023	56013	24372.34	3.01E-56
72 h-H_Cluster 37	Scaffold2366: 1306015–1321028	15014	2510.11	4.19E-59
72 h-H_Cluster 46	Scaffold300: 230249–254907	24659	5437.12	1.42E-64
72 h-H_Cluster 51	Scaffold403: 2–6028	6027	655.50	5.69E-46
72 h-H_Cluster 52	Scaffold4455: 156013–172781	16769	5224.84	3.35E-63
72 h-H_Cluster 60	Scaffold707: 83004–92024	9021	1124.67	1.48E-49
72 h-H_Cluster 63	Scaffold811: 1066000–1071014	5015	615.70	5.94E-42
72 h-H_Cluster 68	Scaffold942: 2627013–2644026	17014	5992.44	5.61E-64
72 h-H_Cluster 70	Scaffold988: 12017–19025	7009	629.34	8.86E-56

*Cluster names are associated with the library name in which the cluster was identified. Induced clusters were expressed only in viruliferous whiteflies, so their expression was considered zero in healthy control whiteflies when edgeR [38] analysis was performed for each time points. Similarly, suppressed clusters were expressed only in healthy control whiteflies, so their expression was considered zero in viruliferous whiteflies.

**Table 5:** Predicted target genes of induced piRNA clusters.

piRNA Cluster*	Target gene	Annotation	Transcripts level in RPKM
24 h-V_Cluster 56	Bta07031	Unknown protein	7.52
48 h-V_Cluster 27	No target gene	N/A	NA
48 h-V_Cluster 42	Bta05801	Unknown protein	5.45
48 h-V_Cluster 57	Bta07186	COMM domain-containing protein 4	4.76
48 h-V_Cluster 57	Bta07187	Sentrin-specific protease 1	0
48 h-V_Cluster 57	Bta07188	Mitochondrial carrier protein	1.5
72 h-V_Cluster 9	Bta02620	Unknown protein	0

*Cluster names are associated with the library name in which the cluster was identified.

N/A indicated that no gene is targeted by a piRNA cluster.

**Table 6:** Transposable elements targeted by induced piRNA clusters.

piRNA Cluster*	DNA Transposon	Retrotransposon
24 h-V_Cluster 56	MITE, hAT, TcMar	LINE/Penelope, LINE/I
48 h-V_Cluster 27		LINE/Jockey, LTR/Gypsy
48 h-V_Cluster 42	MITE, TcMar	LINE/Phenelope
48 h-V_Cluster 57		LINE/R1
72 h-V_Cluster 9		LTR/Gypsy, LTR/Copia

*Cluster names are associated with the library name in which the cluster was identified.

### Suppressed piRNA clusters when whitefly acquired TYLCV

Among those 24 piRNA clusters that were suppressed in whiteflies when fed on TYLCV (Table 4), expression values of the piRNA clusters ranged from 615 to 24,372 RPM, and targeted a total of 17 protein coding genes with diverse biological functions (S6 Table). Eight of them have unknown functions and the remaining genes are not predicted to be involved in any single pathway. In addition to protein coding genes, these piRNA clusters also target both class I and II transposable elements (S7 Table). Class I retrotransposons targeted by these piRNA clusters include LTR and Non-LTR elements. Moreover, a number of class II DNA TE families were also targeted by these piRNA clusters.

## Discussion

piRNAs are widely known to be involved in silencing the activity of transposable elements. Numerous studies on the piRNA pathway have broadened our understanding of the role of piRNA-mediated regulation of TEs in genetic, biological and developmental processes [40–42]. Additionally, piRNAs also regulate the activity of protein coding genes reported for a number of insect species. More recently different groups have reported that piRNAs target the non-coding sequences as well [8,13]. We found that the highest percentage of piRNAs in whitefly is derived from non-coding sequences (Fig 1B), which makes it challenging to infer their potential functions. However, transposable elements and protein coding genes are also targeted by piRNAs.

It was quite interesting to understand that piRNA clusters could be derived either from a positive or from a negative stranded DNA in the whitefly genome. Generation of different orientation of piRNAs was likely due to the two different mechanisms of piRNA biogenesis [7,13,14]. First, piRNA precursors are transcribed from piRNA clusters, and then processed into primary piRNAs [8,16,17], which lead to piRNA-directed cleavage of mRNAs [8,14]. On the other hand, secondary piRNAs are generated through the ping-pong cycle [13,14], which lead to an amplification of piRNA regeneration. The percentage of sense strand-derived piRNAs gradually decreased over time, from 60% at 24 h to 49% at 72 h post virus acquisition in the viruliferous whiteflies, whereas the antisense strand-derived piRNAs increased from 40% to 51% during the same period of time. Although such trends are interesting, we are still unsure about their implications. Recent studies have demonstrated the adaptive antiviral immunity in the mosquito *A*. *aegyti* [43] as well as many of other arthropods [44] suggesting likely involvement of piRNAs in facilitating TYLCV transmission by the vector whitefly *B*. *tabaci*.

In this study, we found that piRNAs were derived from both class I retrotransposons and class II DNA transposons. For class I, three classes of LTR retrotransposons, gypsy, copia and pao-Bel are the most prevalent sources of piRNAs (S4 Table). Previous studies in mosquito and other insects showed the same dominant sources for LTR retrotransposon-derived piRNAs as observed in whitefly [7,11,23]. We also found piRNAs derived from non-LTR retrotransposons, LINEs and SINEs in whitefly. In addition to class I retrotransposons, our study identified piRNAs derived from different families of DNA transposons (S4 Table), including MITE, hAT, TcMar, Maverick, Academ and MULE-MuDR. The highest percentage of piRNAs are derived from MITE DNA transposons when compared among individual TE families (Fig 2). Overall, our results demonstrated that TE-piRNA production in whitefly shared the trends of piRNA biogenesis in other insect species. The mechanism of piRNA-mediated silencing is complex and likely depends on both transcriptional and posttranscriptional mechanisms. Perhaps the mechanism of silencing might differ among different families of transposable elements.

An increasing number of studies reported that piRNAs also target protein coding genes and silence their expression [8,13]. In addition to CDS, they can also target introns and UTRs. Even though gene-derived piRNAs were predominantly mapped to the sense strand in several mosquito species [7,13], we found piRNAs derived from genes in whiteflies are produced from both sense and antisense strands. We observed that the piRNA targeted genes (S3 Table) were not associated with any particular pathway, but instead, seemed to be involved in diverse biological processes.

Our study identified a large number of piRNA clusters. We divided the piRNA clusters into three different categories such as common piRNA clusters, and induced and suppressed piRNA clusters in the presence of TYLCV in whiteflies. We identified 53 common piRNA clusters that are expressed in whiteflies that fed on tomatoes with or without TYLCV infection (S5 Table). These piRNA clusters showed consistent expression across all treatments and did not show any differential expression and targeted numerous classes of transposable elements and protein coding genes of different biological functions. It is likely that these common piRNA clusters are not involved in the acquisition, circulation or transmission of TYLCV in whitefly *B*. *tabaci*.

In addition to common clusters, it was interesting to observe that five piRNA clusters were induced when whiteflies fed on TYLCV-infected tomato. However, the expression of these specific piRNA clusters was not consistent across three time points, suggesting a potential influence from the virus circulation or trafficking through different orgasm in the viruliferous insect vector as measured post acquisition for 24, 48 and 72 h. These specific piRNA clusters targeted six protein coding genes (Table 5). We found that half of the genes have unknown functions, while the other three were annotated as encoding a COMM domain-containing protein, a sentrin-specific protease, and a mitochondrial carrier protein. Based on our previous published transcriptome data [31], these genes are expressed at very low levels, as they are potentially targeted by piRNAs. However, it is still not clear how these proteins are involved in virus transmission or anti-viral immunity in whitefly. The COMM domain family is a multifunctional protein, which could regulate transcription factor NF-kappa-B and copper metabolism. It has been reported that COMM domain-containing proteins modulate the activity of cullin-RING E3 ubiquitin ligase (CRL) complexes [45], and promote the ubiquitination and proteasomal degradation of cellular proteins. Perhaps silencing the expression of this gene in whitefly by piRNAs facilitates deubiquitination of certain cellular proteins involved in virus transmission. Other induced piRNA clusters target sentrin-specific protease 1 (SENP1), which is involved in the small ubiquitin-like modifier (SUMO) pathway. This protein is associated with deconjugation of SUMO from targeted proteins [46,47]. As SENP1 expression is suppressed by the action of piRNA, it could facilitate the conjugation of SUMO to the target proteins, suggesting that piRNA mediated suppression of SENP1 might be playing a role in anti-viral defense, thus to facilitate circulation or trafficking of the virus particles in a viruliferous insect vector. Another gene that was suppressed by the induced piRNA clusters upon acquisition of TYLCV was the mitochondrial carrier protein gene. Mitochondrial carrier proteins transfer molecules across the membranes in the mitochondria. Suppression of the gene encoding mitochondrial carrier protein by a piRNA cluster may facilitate virus trafficking across the membrane to enhance virus transmission. We also looked into the transposable elements targeted by these piRNA clusters. We found that both classes of TEs were targeted by piRNAs, including Class I: LTR/Gypsy, Copia and LINEs/Penelope, R1, L1, and Jockey and class II DNA transposons: MITE, hAT, and TcMar (Table 6). Future in-depth studies are needed to understand the role of piRNAs during virus transmission and immunity.

We also identified 24 piRNA clusters that were induced in whiteflies fed on uninfected tomato (Table 4). There were 15 genes targeted by these piRNA clusters (S6 Table), with one-third of them having unknown functions. The remaining genes were not associated with any particular pathway. Our analysis also found that both class I and II transposable elements were targeted by these piRNA clusters (S7 Table). Class I retrotransposons that were targeted include LTR/Gypsy, Copia, Pao, LINE/DRE, Jockey, Penelope, LOA, CR1, RTE, Dong-4, and SINE, whereas class II DNA transposons include MITE, DNA/Academ, Kolobok-T2, Mariner, MULE-MuDR, Maverick, and hAT. There were multiple members of each of those families of transposable elements. Further studies are needed to pinpoint how the suppression of these piRNAs by TYCLV is associated with anti-viral defense mechanisms. Moreover, a detailed follow up functional study is also required to elucidate the mechanisms on how piRNAs are associated with virus transmission and anti-viral defense in whitefly.

This study provides genome-wide profiling of piRNAs in whiteflies. We found that piRNA clusters were distributed across the whitefly genome, with more than 60% of identified piRNA clusters being derived from non-coding regions with unknown function. Comparative analysis revealed that feeding on a virus-infected host caused induction and suppression of only a small number of piRNA clusters in whiteflies. Majority of the identified piRNA clusters were commonly expressed across all time points in whiteflies fed on TYLCV-infected or uninfected tomato. Apart from piRNAs targeting non-coding regions, other piRNAs target protein coding genes and transposable elements. Although piRNAs primarily regulate the activity of TEs, our results suggest that they may have additional functions in regulating protein coding genes and in insect-virus interactions. This report on piRNA profiling of whitefly will serve as a valuable resource to the whitefly research community. Additionally, this study could also open new avenues to target piRNA-associated genes in whitefly using RNAi or genome editing technologies for vector management.

## Supporting information

S1 Fig Distribution of piRNAs in different genomic regions. Representative piRNA clusters identified by the proTRAC program. It provides detailed information about the piRNA clusters including the genomic coordinates, read coverage, genes and repeat elements falling within the cluster.
(TIFF)

S2 Fig Distribution of piRNAs in different genomic regions. Percentage of piRNAs mapping to different genomic features. Red color denotes piRNAs derived from coding sequences (CDS), green color denotes piRNAs derived from repeat elements, and blue denotes piRNAs derived from non-coding sequences including intergenic regions, introns and UTRs. 24 h-H, 48 h-H, and 72 h-H represents whiteflies fed on uninfected tomato for 24, 48 and 72 hours respectively. 24 h-V, 48 h-V, and 72 h-V represents whiteflies fed on TYLCV-infected tomato for 24, 48 and 72 hours, respectively.
(TIFF)

S1 Table Summary of reads in 18 small RNA libraries. (DOCX)

S2 Table Pearson's correlation coefficients for three biological replicates and Clusters of piRNAs at each of the three different time points and treatments. (XLSX)

S3 Table Predicted piRNAs target genes involved in diverse growth, developmental and metabolic pathways. (XLSX)

S4 Table piRNAs targeting different classes of repeat elements. (XLSX)

S5 Table piRNA clusters commonly expressed across all time points in whiteflies fed on TYLCV-infected or uninfected tomato. (XLSX)

S6 Table Predicted genes targeted by suppressed piRNA clusters. (DOCX)

S7 Table Transposable elements targeted by suppressed piRNA clusters. (DOCX)

## References

[1] Bartel DP. MicroRNAs: Genomics, biogenesis, mechanism, and function. Cell. 2004 pp. 281–297. 10.1016/S0092-8674(04)00045-5 14744438

[2] Ghildiyal M, Zamore PD. Small silencing RNAs: An expanding universe. Nature Reviews Genetics. 2009 pp. 94–108. 10.1038/nrg2504 19148191 PMC2724769

[3] Kim VN, Han J, Siomi MC. Biogenesis of small RNAs in animals. Nature Reviews Molecular Cell Biology. 2009 pp. 126–139. 10.1038/nrm2632 19165215

[4] Aravin AA, Lagos-Quintana M, Yalcin A, Zavolan M, Marks D, Snyder B, et al The small RNA profile during *Drosophila melanogaster* development. Developmental Cell. 2003 pp. 337–350. 10.1016/S1534-5807(03)00228-4 12919683

[5] Malone CD, Hannon GJ. Small RNAs as guardians of the genome. Cell. 2009 pp. 656–668. 10.1016/j.cell.2009.01.045 19239887 PMC2792755

[6] van Rij RP, Berezikov E. Small RNAs and the control of transposons and viruses in *Drosophila*. Trends in Microbiology. 2009 pp. 163–171. 10.1016/j.tim.2009.01.003 19299135

[7] Biryukova I, Ye T. Endogenous siRNAs and piRNAs derived from transposable elements and genes in the malaria vector mosquito *Anopheles gambiae*. BMC Genomics. 2015;16 10.1186/s12864-015-1436-1 25879960 PMC4423592

[8] Liu P, Dong Y, Gu J, Puthiyakunnon S, Wu Y, Chen XG. Developmental piRNA profiles of the invasive vector mosquito *Aedes albopictus*. Parasites and Vectors. 2016;9: 1–15. 10.1186/s13071-015-1291-6 27686069 PMC5041409

[9] Kim VN. Small RNAs just got bigger: Piwi-interacting RNAs (piRNAs) in mammalian testes. Genes and Development. 2006 pp. 1993–1997. 10.1101/gad.1456106 16882976

[10] Zheng K, Xiol J, Reuter M, Eckardt S, Leu NA, McLaughlin KJ, et al Mouse MOV10L1 associates with Piwi proteins and is an essential component of the Piwi-interacting RNA (piRNA) pathway. Proc Natl Acad Sci. 2010;107: 11841–11846. 10.1073/pnas.1003953107 20534472 PMC2900664

[11] Wang W, Ashby R, Ying H, Maleszka R, Forêt S. Contrasting sex-and caste-dependent piRNA profiles in the transposon depleted haplodiploid honeybee *Apis mellifera*. Genome Biol Evol. 2017;9: 1341–1356. 10.1093/gbe/evx087 28472327 PMC5452642

[12] Thomson T, Lin H. The biogenesis and function of PIWI proteins and piRNAs: Progress and prospect. Annu Rev Cell Dev Biol. 2009;25: 355–376. 10.1146/annurev.cellbio.24.110707.175327 19575643 PMC2780330

[13] George P, Jensen S, Pogorelcnik R, Lee J, Xing Y, Brasset E, et al Increased production of piRNAs from euchromatic clusters and genes in *Anopheles gambiae* compared with *Drosophila melanogaster*. Epigenetics and Chromatin. 2015;8 10.1186/s13072-015-0041-5 26617674 PMC4662822

[14] Brennecke J, Aravin AA, Stark A, Dus M, Kellis M, Sachidanandam R, et al Discrete small RNA-generating loci as master regulators of transposon activity in *Drosophila*. Cell. 2007;128: 1089–1103. 10.1016/j.cell.2007.01.043 17346786

[15] Arensburger P, Hice RH, Wright JA, Craig NL, Atkinson PW. The mosquito *Aedes aegypti* has a large genome size and high transposable element load but contains a low proportion of transposon-specific piRNAs. BMC Genomics. 2011;12 10.1186/1471-2164-12-606 22171608 PMC3259105

[16] Voigt F, Reuter M, Kasaruho A, Schulz EC, Pillai RS, Barabas O. Crystal structure of the primary piRNA biogenesis factor Zucchini reveals similarity to the bacterial PLD endonuclease Nuc. RNA. 2012;18: 2128–2134. 10.1261/rna.034967.112 23086923 PMC3504665

[17] Nishimasu H, Ishizu H, Saito K, Fukuhara S, Kamatani MK, Bonnefond L, et al Structure and function of Zucchini endoribonuclease in piRNA biogenesis. Nature. 2012;491: 284–287. 10.1038/nature11509 23064230

[18] Nagao A, Mituyama T, Huang H, Chen D, Siomi MC, Siomi H. Biogenesis pathways of piRNAs loaded onto AGO3 in the *Drosophila testis*. RNA. 2010;16: 2503–2515. 10.1261/rna.2270710 20980675 PMC2995411

[19] Houwing S, Kamminga LM, Berezikov E, Cronembold D, Girard A, van den Elst H, et al A role for Piwi and piRNAs in germ cell maintenance and transposon silencing in zebrafish. Cell. 2007;129: 69–82. 10.1016/j.cell.2007.03.026 17418787

[20] Morazzani EM, Wiley MR, Murreddu MG, Adelman ZN, Myles KM. Production of virus-derived ping-pong-dependent piRNA-like small RNAs in the *mosquito soma*. PLoS Pathog. 2012;8 10.1371/journal.ppat.1002470 22241995 PMC3252369

[21] Miller WJ, McDonald JF, Pinsker W. Molecular domestication of mobile elements. Genetica. 1997;100: 261–70. 10.1023/A:1018306317836 9440279

[22] Kapitonov V V., Jurka J. A universal classification of eukaryotic transposable elements implemented in Repbase. Nature Reviews Genetics. 2008 pp. 411–412. 10.1038/nrg2165-c1 18421312

[23] Bargues N, Lerat E. Evolutionary history of LTR-retrotransposons among 20 *Drosophila species*. Mob DNA. 2017;8 10.1186/s13100-017-0090-3 28465726 PMC5408442

[24] McCullers TJ, Steiniger M. Transposable elements in *Drosophila*. Mob Genet Elements. 2017;7: 1–18. 10.1080/2159256X.2017.1318201 28580197 PMC5443660

[25] Hess AM, Prasad AN, Ptitsyn A, Ebel GD, Olson KE, Barbacioru C, et al Small RNA profiling of Dengue virus-mosquito interactions implicates the PIWI RNA pathway in anti-viral defense. BMC Microbiol. 2011;11 10.1186/1471-2180-11-45 21356105 PMC3060848

[26] Schnettler E, Donald CL, Human S, Watson M, Siu RWC, McFarlane M, et al Knockdown of piRNA pathway proteins results in enhanced semliki forest virus production in mosquito cells. J Gen Virol. 2013;94: 1680–1689. 10.1099/vir.0.053850-0 23559478 PMC3709635

[27] Navas-Castillo J, Fiallo-Olivé E, Sánchez-Campos S. Emerging virus diseases transmitted by whiteflies. Annu Rev Phytopathol. 2011;49: 219–248. 10.1146/annurev-phyto-072910-095235 21568700

[28] Chen W, Hasegawa DK, Kaur N, Kliot A, Pinheiro PV, Luan J, et al The draft genome of whitefly *Bemisia tabaci* MEAM1, a global crop pest, provides novel insights into virus transmission, host adaptation, and insecticide resistance. BMC Biol. 2016;14 10.1186/s12915-016-0321-y 27974049 PMC5157087

[29] Luan JB, Wang XW, Colvin J, Liu SS. Plant-mediated whitefly-begomovirus interactions: Research progress and future prospects. Bulletin of Entomological Research. 2014 pp. 267–276. 10.1017/S000748531400011X 24548638

[30] Ghanim M, Czosnek H. Interactions between the whitefly *Bemisia tabaci* and begomoviruses: Biological and genomic perspectives. Management of Insect Pests to Agriculture: Lessons learned from deciphering their genome, transcriptome and proteome. 2016 pp. 181–200. 10.1007/978-3-319-24049-7_7

[31] Hasegawa DK, Chen W, Zheng Y, Kaur N, Wintermantel WM, Simmons AM, et al Comparative transcriptome analysis reveals networks of genes activated in the whitefly, *Bemisia tabaci* when fed on tomato plants infected with tomato yellow leaf curl virus. Virology. 2018;513: 52–64. 10.1016/j.virol.2017.10.008 29035786

[32] Vodovar N, Bronkhorst AW, van Cleef KWR, Miesen P, Blanc H, van Rij RP, et al Arbovirus-derived piRNAs exhibit a ping-pong signature in mosquito cells. PLoS One. 2012;7 10.1371/journal.pone.0030861 22292064 PMC3265520

[33] Chen YR, Zheng Y, Liu B, Zhong S, Giovannoni J, Fei Z. A cost-effective method for Illumina small RNA-Seq library preparation using T4 RNA ligase 1 adenylated adapters. Plant Methods. 2012; 10.1186/1746-4811-8-41 22995534 PMC3462708

[34] Li R, Gao S, Hernandez AG, Wechter WP, Fei Z, Ling KS. Deep sequencing of small RNAs in tomato for virus and viroid identification and strain differentiation. PLoS One. 2012; 10.1371/journal.pone.0037127 22623984 PMC3356388

[35] Bolger AM, Lohse M, Usadel B. Trimmomatic: A flexible trimmer for Illumina sequence data. Bioinformatics. 2014;30: 2114–2120. 10.1093/bioinformatics/btu170 24695404 PMC4103590

[36] Langmead B, Trapnell C, Pop M, Salzberg S. Ultrafast and memory-efficient alignment of short DNA sequences to the human genome. Genome Biol. 2009;10: R25 10.1186/gb-2009-10-3-r25 19261174 PMC2690996

[37] Rosenkranz D, Zischler H. proTRAC—a software for probabilistic piRNA cluster detection, visualization and analysis. BMC Bioinformatics. 2012;13 10.1186/1471-2105-13-5 22233380 PMC3293768

[38] Robinson MD, McCarthy DJ, Smyth GK. edgeR: A Bioconductor package for differential expression analysis of digital gene expression data. Bioinformatics. 2009; 10.1093/bioinformatics/btp616 19910308 PMC2796818

[39] Quinlan AR, Hall IM. BEDTools: A flexible suite of utilities for comparing genomic features. Bioinformatics. 2010;26: 841–842. 10.1093/bioinformatics/btq033 20110278 PMC2832824

[40] Lee YCG. The role of piRNA-mediated epigenetic silencing in the population dynamics of transposable elements in *Drosophila melanogaster*. PLoS Genet. 2015;11 10.1371/journal.pgen.1005269 26042931 PMC4456100

[41] Handler D, Meixner K, Pizka M, Lauss K, Schmied C, Gruber FS, et al The genetic makeup of the *Drosophila* piRNA pathway. Mol Cell. 2013;50: 762–777. 10.1016/j.molcel.2013.04.031 23665231 PMC3679447

[42] Simonelig M. Developmental functions of piRNAs and transposable elements: a *Drosophila* point-of-view. RNA Biol. 2011;8: 754–759. 10.4161/rna.8.5.16042 21712652 PMC3256352

[43] Tassetto M, Kunitomi M, Whitfield ZJ, Dolan P, Vargas IS, Ribiero I, Chen T, Olson KE, and Andino R. Antiviral adaptive immunity and tolerance in the mosquito *Aedes aegyti* bioRxiv preprint first posted online Oct. 9, 2018; 10.1101/438911

[44] ter Horst AM, Nigg JC, and Falk BW. Endogenous viral elements are widespread in arthropod genomes and commonly give rise to piRNAs. bioRxiv preprint first posted online Aug. 20, 2018; 10.1101/396382. PMC6401445 30567990

[45] Mao X, Gluck N, Chen B, Starokadomskyy P, Li H, Maine GN, et al COMMD1 (Copper Metabolism MURR1 Domain-containing protein 1) regulates Cullin RING ligases by preventing CAND1 (Cullin-associated Nedd8-dissociated protein 1) binding. J Biol Chem. 2011;286: 32355–32365. 10.1074/jbc.M111.278408 21778237 PMC3173175

[46] Gong L, Millas S, Maul GG, Yeh ET. Differential regulation of sentrinized proteins by a novel sentrin-specific protease. J Biol Chem. 2000;275: 3355–3359. 10.1074/jbc.275.5.3355 10652325

[47] Bailey D, O’Hare P. Characterization of the localization and proteolytic activity of the SUMO-specific protease, SENP1. J Biol Chem. 2004;279: 692–703. 10.1074/jbc.M306195200 14563852

