# Supplementary figures and images for: Genome-wide profiling of piRNAs in the whitefly Bemisia tabaci reveals cluster distribution and association with begomovirus transmission
Source: PLoS One. 2019 Mar 12;14(3):e0213149. doi: 10.1371/journal.pone.0213149 (PMC6413925; doi:10.1371/journal.pone.0213149)

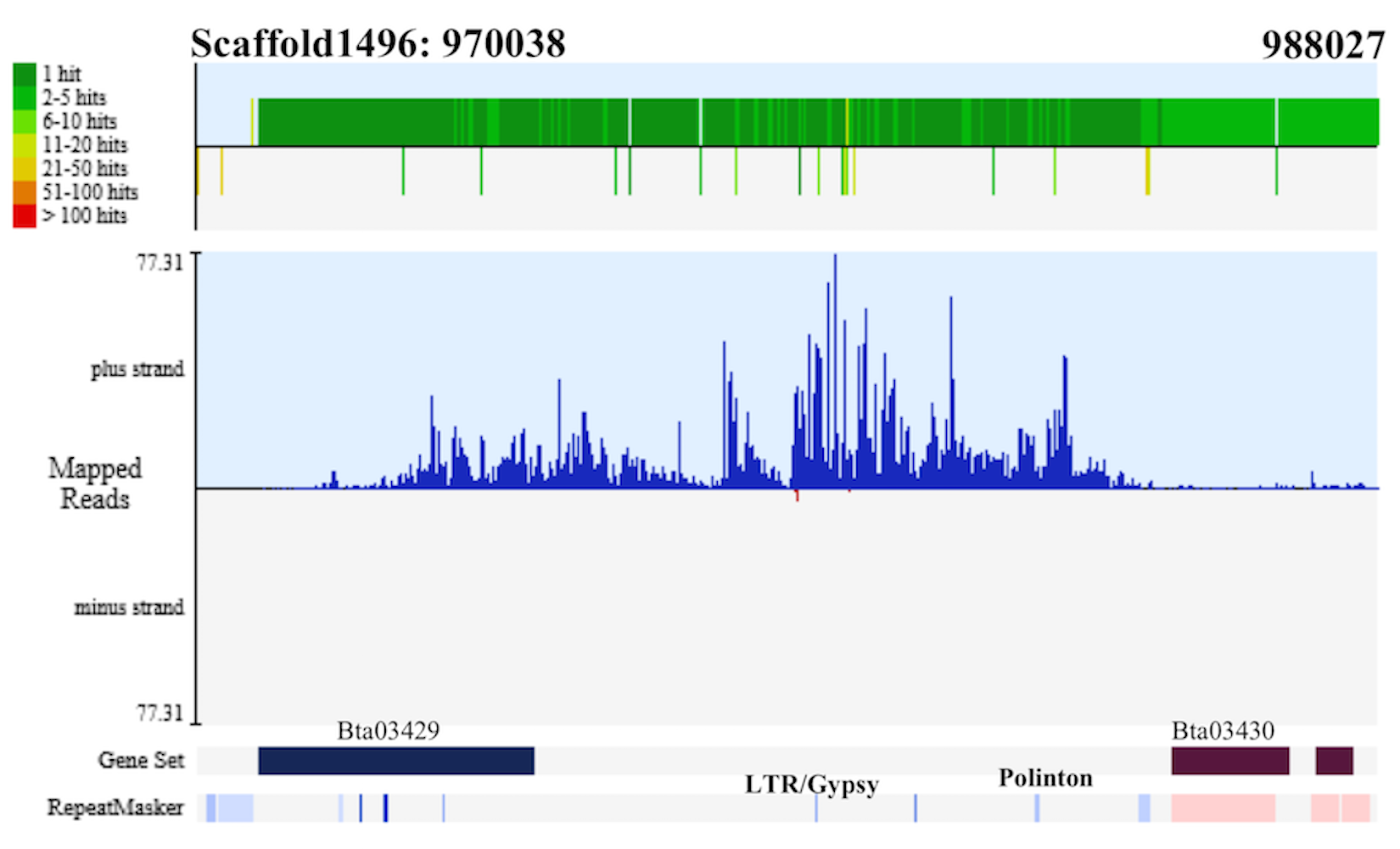

Supplement: S1 Fig — Representative piRNA clusters identified by the proTRAC program. It provides detailed information about the piRNA clusters including the genomic coordinates, read coverage, genes and repeat elements falling within the cluster. (TIFF) [file pone.0213149.s001.tiff]

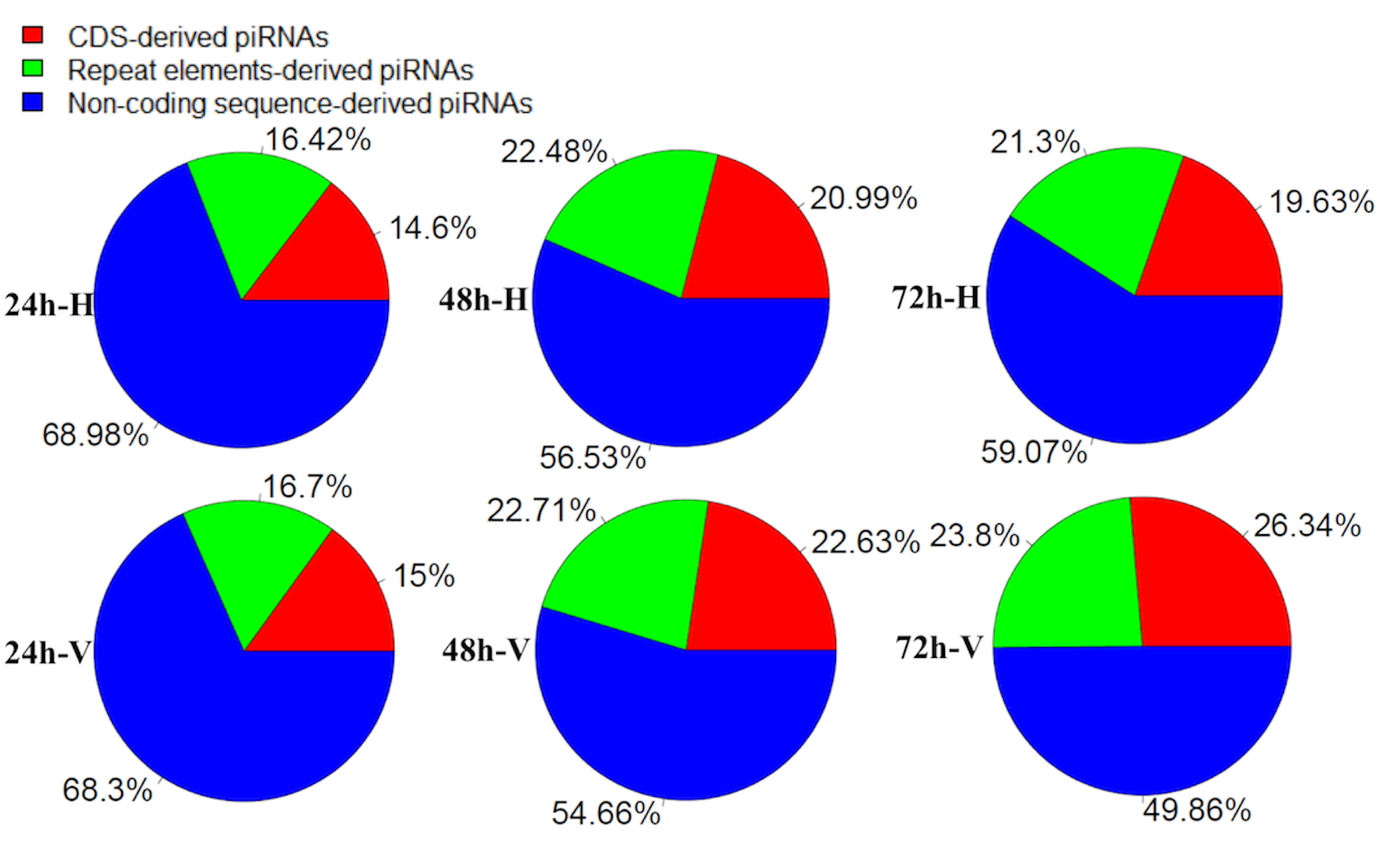

Supplement: S2 Fig — Percentage of piRNAs mapping to different genomic features. Red color denotes piRNAs derived from coding sequences (CDS), green color denotes piRNAs derived from repeat elements, and blue denotes piRNAs derived from non-coding sequences including intergenic regions, introns and UTRs. 24 h-H, 48 h-H, and 72 h-H represents whiteflies fed on uninfected tomato for 24, 48 and 72 hours respectively. 24 h-V, 48 h-V, and 72 h-V represents whiteflies fed on TYLCV-infected tomato for 24, 48 and 72 hours, respectively. (TIFF) [file pone.0213149.s002.tiff]
