# Supplementary material for: Genome-wide profiling of piRNAs in the whitefly Bemisia tabaci reveals cluster distribution and association with begomovirus transmission
Source: PLoS One. 2019 Mar 12;14(3):e0213149. doi: 10.1371/journal.pone.0213149 (PMC6413925; doi:10.1371/journal.pone.0213149)
Supplement: S1 Table — (DOCX) [file pone.0213149.s003.docx]

**S1 Table. Summary of reads in 18 small RNA libraries.**

| **Sample** | **Raw Reads** | **Clean Reads** | **Genome Mapped Reads** | |
| --- | --- | --- | --- | --- |
| 24 h Uninfected rep 1 | 11,396,636 | 6,450,138 | 4,597,965 |  |
| 24 h Uninfected rep 2 | 13,896,556 | 8,000,547 | 5,817,495 |  |
| 24 h Uninfected rep 3 | 10,033,516 | 5,709,591 | 4,069,067 |  |
| 24 h TYLCV rep 1 | 8,496,418 | 5,028,472 | 3,640,965 |  |
| 24 h TYLCV rep 2 | 10,948,319 | 6,554,168 | 4,819,960 |  |
| 24 h TYLCV rep 3 | 9,964,655 | 5,812,253 | 4,293,954 |  |
| 48 h Uninfected rep 1 | 6,568,291 | 3,380,384 | 2,635,534 |  |
| 48 h Uninfected rep 2 | 11,990,650 | 4,989,984 | 3,710,554 |  |
| 48 h Uninfected rep 3 | 9,460,076 | 3,831,989 | 2,872,969 |  |
| 48 h TYLCV rep 1 | 5,571,966 | 1,877,534 | 1,361,023 |  |
| 48 h TYLCV rep 2 | 11,305,333 | 4,649,274 | 3,528,425 |  |
| 48 h TYLCV rep 3 | 10,044,303 | 3,517,535 | 2,688,076 |  |
| 72 h Uninfected rep 1 | 9,339,697 | 4,631,281 | 3,474,041 |  |
| 72 h Uninfected rep 2 | 10,346,125 | 5,245,471 | 3,824,427 |  |
| 72 h Uninfected rep 3 | 7,472,600 | 3,365,760 | 2,361,219 |  |
| 72 h TYLCV rep 1 | 12,380,899 | 5,550,218 | 4,123,349 |  |
| 72 h TYLCV rep 2 | 7,682,783 | 2,845,521 | 2,105,697 |  |
| 72 h TYLCV rep 3 | 8,680,541 | 3,785,570 | 2,764,259 |  |
| Total | 175,579,364 | 85,225,690 | 62,688,979 |  |
