# Supplementary material for: Genome-wide profiling of piRNAs in the whitefly Bemisia tabaci reveals cluster distribution and association with begomovirus transmission
Source: PLoS One. 2019 Mar 12;14(3):e0213149. doi: 10.1371/journal.pone.0213149 (PMC6413925; doi:10.1371/journal.pone.0213149)
Supplement: S6 Table — (DOCX) [file pone.0213149.s008.docx]

**S6 Table. Predicted genes targeted by suppressed piRNA clusters.**

| **piRNA Cluster*** | **Target Gene** | **Annotation** | **Transcripts level in RPKM** |
| --- | --- | --- | --- |
| 24 h-H_Cluster 32 | Bta04318 | Ubiquitin carboxyl-terminal hydrolase protein | 0.11 |
| 24 h-H_Cluster 32 | Bta04319 | Unknown protein | 2.68 |
| 24 h-H_Cluster 39 | No Target Gene | N/A | N/A |
| 24 h-H_Cluster 43 | Bta05610 | Collagen alpha-2(XI) chain | 0.11 |
| 24 h-H_Cluster 43 | Bta05611 | Alk-exo | 0 |
| 24 h-H_Cluster 43 | Bta05612 | Unknown protein | 0 |
| 24 h-H_Cluster 43 | Bta05613 | Unknown protein | 0 |
| 24 h-H_Cluster 49 | Bta05949 | RNA-directed RNA polymerase L | 0 |
| 24 h-H_Cluster 72 | No Target Gene | N/A | N/A |
| 48 h-H_Cluster 21 | No Target Gene | N/A | N/A |
| 48 h-H_Cluster 36 | Bta05946 | Unknown protein | 7.63 |
| 48 h-H_Cluster 38 | No Target Gene | N/A | N/A |
| 48 h-H_Cluster 57 | No Target Gene | N/A | N/A |
| 72 h-H_Cluster 3 | No Target Gene | N/A | N/A |
| 72 h-H_Cluster 9 | No Target Gene | N/A | N/A |
| 72 h-H_Cluster 13 | No Target Gene | N/A | N/A |
| 72 h-H_Cluster 17 | No Target Gene | N/A | N/A |
| 72 h-H_Cluster 22 | Bta04318 | Ubiquitin carboxyl-terminal hydrolase protein | 0.1 |
| 72 h-H_Cluster 22 | Bta04319 | Unknown protein | 2.35 |
| 72 h-H_Cluster 30 | Bta05450 | THAP domain-containing protein 11 | 0.74 |
| 72 h-H_Cluster 36 | Bta05946 | Unknown protein | 9.16 |
| 72 h-H_Cluster 37 | No Target Gene | N/A | N/A |
| 72 h-H_Cluster 46 | Bta07715 | Exostosin-3 | 19.13 |
| 72 h-H_Cluster 51 | Bta10262 | DNA Pol B2 domain-containing protein | 0.31 |
| 72 h-H_Cluster 52 | Bta10540 | Zinc finger protein 76 | 4.08 |
| 72 h-H_Cluster 60 | No Target Gene | N/A | N/A |
| 72 h-H_Cluster 63 | Bta14658 | Unknown protein | 0 |
| 72 h-H_Cluster 68 | Bta15319 | Unknown protein | 7.06 |
| 72 h-H_Cluster 70 | No Target Gene | N/A | N/A |

*Cluster names are associated with the library name in which the cluster was identified.

N/A indicates that no gene is targeted by a piRNA cluster.
