# Supplementary material for: Genome-wide profiling of piRNAs in the whitefly Bemisia tabaci reveals cluster distribution and association with begomovirus transmission
Source: PLoS One. 2019 Mar 12;14(3):e0213149. doi: 10.1371/journal.pone.0213149 (PMC6413925; doi:10.1371/journal.pone.0213149)
Supplement: S7 Table — (DOCX) [file pone.0213149.s009.docx]

**S7 Table. Transposable elements targeted by suppressed piRNA clusters.**

| **piRNA Cluster*** | **DNA Transposon** | **Retrotransposon** |
| --- | --- | --- |
| 24 h-H_Cluster 32 | DNA/Academ |  |
| 24 h-H_Cluster 39 |  | LTR/Gypsy, LINE/Jockey |
| 24 h-H_Cluster 43 | DNA/Kolobok-T2 |  |
| 24 h-H_Cluster 49 | MITE | LTR/Gypsy, LINE/DRE, L1 |
| 24 h-H_Cluster 72 | DNA/Academ, DNA/Mariner | LTR/Pao |
| 48 h-H_Cluster 21 |  | LINE/Jockey |
| 48 h-H_Cluster 36 | DNA/MULE-MuDR, MITE | LTR/Pao, LINE/CR1, L1, Dong-R4 |
| 48 h-H_Cluster 38 | MITE | LINE/CR1 |
| 48 h-H_Cluster 57 | MITE, DNA/Maverick |  |
| 72 h-H_Cluster 3 | MITE | LTR/Gypsy, LINE/Jockey, RTE |
| 72 h-H_Cluster 9 | MITE | LINE/Penelope |
| 72 h-H_Cluster 13 | MITE |  |
| 72 h-H_Cluster 17 | MITE | LINE/LOA, R1 |
| 72 h-H_Cluster 22 | DNA/Academ |  |
| 72 h-H_Cluster 30 |  | LTR/Gypsy |
| 72 h-H_Cluster 36 | DNA/MULE-MuDR, MITE | LTR/Pao, LINE/CR1, L1, Dong-R4 |
| 72 h-H_Cluster 37 | MITE | LINE/Jockey |
| 72 h-H_Cluster 46 | MITE | LTR/Copia, LINE/Penelope |
| 72 h-H_Cluster 51 |  |  |
| 72 h-H_Cluster 52 | MITE, DNA/hAT | LTR/Gypsy, LINE/Jockey, I, R1 |
| 72 h-H_Cluster 60 |  | LTR/Pao, LINE/Jockey,L1 |
| 72 h-H_Cluster 63 |  | LTR/Gypsy |
| 72 h-H_Cluster 68 | MITE | LINE/Dong-R4, SINE |
| 72 h-H_Cluster 70 | MITE |  |

*Cluster names are associated with the library name in which the cluster was identified
